# Family history of colorectal cancer in Iran

**DOI:** 10.1186/1471-2407-5-112

**Published:** 2005-09-05

**Authors:** Mahboobeh Mahdavinia, Faraz Bishehsari, Reza Ansari, Nasim Norouzbeigi, Ahmad Khaleghinejad, Mahshid Hormazdi, Naser Rakhshani, Reza Malekzadeh

**Affiliations:** 1 Digestive Disease Research Centre (DDRC), Shariati Hospital, Tehran University of Medical sciences, Tehran, Iran; 2 General surgery ward, Mehr Hospital, Tehran, Iran; 3 Pathology Department, Mehr Hospital, Tehran, Iran

## Abstract

**Background:**

Previous reports show a high proportion of young CRC patients in Iran. In this study we aim to look for the clustering of colorectal cancer in families of a series of CRC patients from Iran.

**Methods:**

The family history of cancer is traced in 449 CRC patients of which 112 were 45 yrs or younger and 337 were older than 45 yrs at time of diagnosis. The patients were admitted in two hospitals in Tehran, during a 4-year period.

**Results:**

Clinical diagnosis of HNPCC was established in 21 (4.7%) probands. Family history of CRC was more frequently reported by early-onset than by late-onset patients (29.5% vs. 12.8%, p < 0.001).

Distribution of tumor site differed significantly between those with and without family history of CRC. Right colon cancer was the most frequent site (23/45, 35.4%) observed in patients with positive family history of colorectal cancer.

**Conclusion:**

The relatively high frequency of CRC clustering along with HNPCC in our patients should be further confirmed with larger sample size population-based and genetic studies to establish a cost effective molecular screening for the future.

## Background

Colorectal carcinoma (CRC) is the third most common cause of cancer related deaths in the world [1] with some well known hereditary forms. Hereditary non-polyposis colorectal cancer (HNPCC) is the most common type of hereditary CRC. The frequency of HNPCC varies worldwide ranging from 1 to 6% [2]. This variability could be due to discrepancies in definitions (clinical *vs*. molecular criteria) and also regional heterogeneity [3-5].

Apart from defined genetic syndromes of CRC, both retrospective and prospective studies proved that familial history of colorectal cancer could increase a person's lifetime risk of colorectal cancer significantly [6,7].

CRC with age-adjusted rate of 6–7.9 per 100,000 person per year is the fourth most common cancer in Iran [8,9]. Early-onset colorectal cancer (less than 40 yrs of age at time of diagnosis) comprises almost one fifth of all CRC cases in the country [9,10]. This proportion is considerably lower in high-risk countries, with rates ranging from 2% to 8% [11,12]. The high proportions of young CRC cases seen in Iran, and probably many neighboring countries, are mainly due to the young age-structure of these countries and relatively low rates of CRC in older individuals [10].

Up to now there have been no reports addressing the familial aggregation of colorectal cancer in Iran. In the current study, we aimed to study the clustering of colorectal cancer in families of CRC patients and make a comparison of frequency of colorectal and other types of cancer in family members of early-onset with that of late-onset CRC patients.

## Methods

We reviewed all patients' documents with a pathologically confirmed diagnosis of colorectal adenocarcinoma admitted in two hospitals in Tehran between January 2000 and February 2004. Patients with familial adenomatous polyposis or those with underlying inflammatory bowel disease were excluded. Of 662 patients registered in the database, 174 patients were lost due to change of address or contact phone number, 33 refused to be interviewed and 6 were excluded because of incomplete family history accordingly, 449 CRC cases (112 younger than 45 yrs and 337 older than 45 yrs at diagnosis) or their close family members participated in this study.

Demographic, clinical and tumor-related characteristics of patients were recorded based on their hospital documents. These parameters included gender, age at diagnosis, place and date of birth and tumor-related factors such as location, stage, degree of differentiation and mucus production.

All of these patients or their siblings/parents (in the case the patient was dead or not available) were interviewed to trace their family history of cancer including occurrence of malignancy in the family, type of cancer and the age at diagnosis of the affected family member. Pedigrees were drawn at least up to second-degree relatives. The obtained pedigrees were reconfirmed by interviewing another member of family. In addition, we tried to verify reported malignancies in relatives by asking for their medical records, if available. In Iran, family relations are very strong and people are usually aware of serious diseases such as cancer in their relatives.

Patients belonging to families fulfilling the AmsterdamII criteria, including at least three members with an HNPCC-associated cancer (colorectal, endometrial, small bowel, ureter, renal pelvis) in at least two successive generations, one being a first-degree relative of the other two and at least one diagnosed before the age of 50 years, were classified as HNPCC [13].

Patients from families which did not fulfill Amsterdam criteria but with at least two relatives with colorectal cancer in a first- or second-degree relationship were classified as Hereditary Colorectal Cancer (HCRC).

This study was approved by the Digestive Disease Research Center of Tehran University of Medical Sciences Institutional Review Board and informed consents were obtained from patients or their families participating in the study.

### Statistical analysis

Qualitative variables were compared by chi-squared test with Yates correction when needed. A *P *value of less than 0.05 was considered to indicate a statistically significant difference. All calculations were performed using the 11.5 SPSS software package (SPSS Inc., Chicago, IL, USA).

## Results

Four-hundred forty nine (449) CRC patients with pathologically confirmed colorectal adenocarcinoma were enrolled in the study.

Tumor sites of early-onset and late-onset group are separately shown in table 1. No significant difference in localization of tumor was observed between the two groups.

Family history of cancer up to second-degree relatives were observed in 60 (53.5%) of early-onset and 144 (43.5%) of late-onset patients respectively. The most common cancers affecting first and second degree relatives are shown in table 2. History of colorectal cancer in at least one relative was significantly more frequent in early-onset patients; 33 (29.5%) comparing to 43 (12.8%) cases in late-onset group. Frequency of other types of cancers reported in the family did not differ significantly between the two age groups.

The frequency of hereditary types of colorectal cancer in two age groups is shown in Table 3. Among 21 HNPCC families, nineteen fulfilled the Amsterdam criteria I having at least three members with CRC in two consecutive generations, one being a first-degree relative of the other two and at least one diagnosed before the age of 50 years [14]. The other 2 families fulfilled the Amsterdam criteria II with history of colorectal and endometrial cancer in the family.

Distribution of tumor site differed significantly between patients with family history of CRC and those without this history (Table 4,†); In total, right colon cancer was seen more frequently than other locations in probands with family history of CRC, in contrast to higher percentage of left colon carcinoma in patients without this history.

Rectal carcinoma was rarely seen in young patients with positive family history; 12.1% comparing to 38% in those without family history (p = 0.025) or cases belonging to the other age group.

## Discussion

This report represents the first study to characterize the profile of familial CRC aggregation in Iranian patients. Although we tried to include all recorded patients in this study, familial profile of 213 (32%) cases could not be determined and analyzed here (see method). However, there were no significant differences in age, sex and tumor localization of participating and non participating patients.

The estimation of the frequency of HNPCC among CRC patients based on family history varies in different populations. In a multicenter study on Finnish population, the frequency of families meeting the Amsterdam criteria was 1.7% [15]. Ponz et al estimated the frequency of HNPCC among CRC cases to be 3.4–4.5% in Northern Italy [3], while in Spanish population; clinical diagnosis of HNPCC was established in 2.5% of CRC patients [16]. In a pilot study by Soliman a et al, 7.2% of Egyptian colorectal patients had family history suggestive of hereditary non polyposis colorectal cancer [17]. Discrepancies in the reported frequency of HNPCC between populations probably reflect population differences. Frequency of this syndrome in Iran has not yet been studied. In our study, a total of 21 cases (4.7%) met the Amsterdam criteria II. This relatively high frequency of HNPCC in our patients should be further confirmed with larger sample size, population-based, and genetic studies.

HNPCC clinical diagnosis was significantly more prevalent among younger patients in agreement with previous studies [18,19]. In some other studies, the mean age at diagnosis for HNPCC patients was reported to be between 52–60 yrs [20,21]. However, we found that almost 3% of patients over 45 yrs belonged to families fulfilling Amsterdam criteria. This indicates that taking detailed family history even in old cases is necessary and could identify CRC families with known genetic risk.

Also, we have found higher frequency of familial clustering of CRC apart from HNPCC cases in younger patients (Table 3).This in agreement with studies in other populations, suggests stronger genetic background in younger onset CRC patients [22].

We observed 15 families who did not fulfill HNPCC criteria, but with more than 2 CRC relatives in the family (Table 3). It was previously suggested that some familial cancers might be due to common environmental exposures of family members rather than genetic clustering of cancer [23], but more recently; it has been shown that familial risk for CRC are mainly due to heritable causes [24]. Therefore, these families could be added to HNPCC families to undergo analysis of MMR genes [25]. However, not all CRC clustering could be attributed to defects in genes involved in mismatch repair [26], and unknown loci may be responsible for much of the familial aggregation of CRC [27].

It is suggested that family history of CRC is related to the localization of tumor. Some studies proposed a stronger familial component for proximal than for distal colon cancer [6,28], while this association was not observed in other studies [29]. In our study, right-sided tumors occurred more frequently in patients with positive family history of CRC compared to those without this history (36.9% vs. 17.7%, p < 0.001). This pattern was almost the same in patients over and under 45 yrs of age (Table 4). Also, we have found rectal cancer to be less frequent among younger probands with positive CRC family history. This is in agrremnet with the previous report of Olsson L and Lindblom A, in which the frequency of sigmoid cancer was shown to be lower among familial CRC cases compared to sporadic patients [21]. Altogether, these findings could indicate a difference in carcinogenic pathways based on the tumor location with heritable causes mostly affecting the right colon and exogenous factors responsible for carcinogenesis in the distal part of the large bowel. It has been suggested that chromosomal instability affecting allelic losses of APC and P53 are more frequent in distal colonic and rectal tumors than in proximal lesions. On the other hand, microsatellite instability is more common in proximal tumors [30].

Appropriate screening strategies should be considered to decrease the burden of CRC in Iran.Our ongoing molecular and genetic studies including analysis of micro satellite instability and mismatch repair gene mutations would most likely help us to characterize the molecular basis for the observed clustering of colorectal cancer among families of younger onset patients and could lead to the adoption of simpler and more cost effective molecular screening in the future.

## Conclusion

Clinical diagnosis of HNPCC was observed in 21 (4.7%) probands in our study. Familial clustering of CRC was more frequent in younger probands. The right side of the colon was more frequently affected in patients with positive family history of CRC. This indicates that a detailed family history is mandatory in colorectal cancer patients with particular attention to younger onset cases and those with right-sided tumors.

## Authors' contributions

MM and FB designed and conducted the study, interviewed the patients, analyzed the data, and drafted the manuscript.

RA, NN and AK assisted in conducting the study and interviewing the patients.

MH and NR reviewed and approved the pathology reports of the patients.

RM supervised the study scientifically, has been involved in designing the study and preparing the manuscript and revising it for scientific content and has given final approval of the version to be published.

All authors read and approved the final manuscript.

## Pre-publication history

The pre-publication history for this paper can be accessed here:



## Figures and Tables

**Table 1:** CRC characteristics among young and old patients.

Variable	Early-onset (n = 112)	Late-onset (n = 337)	P Value^†^
Sex (M/F)	43/69	138/199	NS^††^
Tumor Location			
Right	30(26.8%)	62(18.4%)	NS
Left	43(38.4%)	134(39.8%)	NS
Rectum	36(32.1%)	141(41.8%)	NS
NOS	3(2.6%)	0	___

†Assigns the significance of the differences between two group

†† NS: Not significant

**Table 2:** Frequent cancer sites in relatives of early and late-onset CRC patients.

	Early-onset (n = 112)	Late-onset (n = 337)	P Value^†^
Family History (FHx) of cancer	60(53.6%)	144(42.7%)	NS^††^
FHx of CRC	33(29.4%)	43(12.8%)	<0.001
Breast	11(9.8%)	17(5%)	NS
Stomach	9(8%)	17(5%)	NS
Lung	5(4.5%)	25(7.5%)	NS
Leukemia & Lymphoma	4(3.6%)	14(4.2%)	NS
Brain	4(3.6%)	9(2.7%)	NS
Endometrial	2(1.8%)	12(3.6%)	NS

†Assigns the significance of the differences between two group

†† NS: Not significant

**Table 3:** Hereditary forms of CRC in two age groups.

Variable	Total (n = 449)	Early-onset (n = 112)	Late-onset (n = 337)	P Value^†^
HNPCC	21(4.7%)	12(10.7%)	9(2.9%)	<0.001
HCRC	15(3.4%)	9(8%)	6(1.7%)	<0.001
One first-degree relative with CRC	26(5.8%)	7(6%)	19(5.6%)	NS^††^

†Assigns the significance of the differences between two group

†† NS: Not significant

**Table 4:** distribution of tumor sites by family history of colorectal cancer according to age of the proband.

Age group	Positive FH^† ^of CRC	Negative FH of CRC
CRC localization		
Total^†^	N = 76	N = 373
Right^a^	28(36.9%)	66(17.7%)
Left	24(31.6%)	167(44.8%)
Rectum	22(29%)	139(37.3%)
NOS	2(2.5%)	1(0.03%)
Over 45	N = 43	N = 294
Right^b^	14(32.6%)	49(16.7%)
Left	11(25.6%)	136(46.3%)
Rectum	18(41.8%)	109(37.1%)
Less than 45	N = 33	N = 79
Right^c^	14(42.4%)	17(21.5%)
Left	13(39.4%)	31(39.2%)
Rectum	4(12.1%)	30(38%)
NOS	2(6.1%)	1(1.3%)

†: difference of tumor site distribution between two groups (p < 0.001)

a,b,c: p < 0.05
